# Regional hyperthermia combined with radiotherapy for esophageal squamous cell carcinoma with supraclavicular lymph node metastasis

**DOI:** 10.18632/oncotarget.14148

**Published:** 2016-12-24

**Authors:** Sheng Liming, Ji Yongling, Wu Qiner, Du Xianghui

**Affiliations:** ^1^ Department of Radiotherapy, Zhejiang Cancer Hospital, Hangzhou, Zhejiang, China; ^2^ Key Laboratory Diagnosis and Treatment Technology on Thoracic Oncology, Zhejiang, China; ^3^ Department of Hyperthermia, Zhejiang Cancer Hospital, Hangzhou, Zhejiang, China

**Keywords:** esophageal cancer, regional hyperthermia, radiotherapy, prognosis

## Abstract

To assess the efficacy and toxicity of Intensity-modulated radiotherapy (IMRT) and hyperthermia for upper and middle thoracic esophageal squamous cell carcinoma (UMT-ESCC) with supraclavicular lymph node metastasis. A total of 50 patients with UMT-ESCC with supraclavicular lymph node metastasis were evaluated in this retrospective study. All patients received IMRT. Hyperthermia was delivered simultaneously with irradiation, in 45 minutes twice a week for 5-6 weeks. Hyperthermia included supraclavicular lymph node metastasis. Forty-four patients (88.0%) received concurrent chemoradiotherapy based on cisplatin regimens. The most common types of hematological toxicities were anemia (62.0%) and leukopenia (60.0%). Most of these events were grade 1-2 and transient. The 3-year progression-free survival (PFS) rate and overall survival (OS) rate were 34.9% and 42.5%, respectively. Cox regression revealed that tumor length and number of supraclavicular lymph node metastasis were two independent predictors of OS (tumor length: HR=3.65, p=0.008; nodal stage: HR=8.07, p=0.019). The IMRT combined with supraclavicular regional hyperthermia has low toxicity and well tolerated with excellent local control in UMT-ESCC with supraclavicular lymph node metastasis.

## INTRODUCTION

Esophageal squamous cell carcinoma (ESCC) is the leading cancer-related cause of death in China. Approximately 8-20% of these patients have supraclavicular lymph node metastasis [1–3]. The prognosis of upper and middle thoracic esophageal squamous cell carcinoma (UMT-ESCC) with supraclavicular lymph node metastasis is still unsatisfactory, with 5-year survival rate 20% [4]. Radiotherapy plays important role in the treatment of locally advanced ESCC. The development of technologies such as intensity-modulated radiotherapy (IMRT) and image-guided radiotherapy (IGRT) permitted to deliver radiation doses to tumor while sparing the adjacent organ-at-risk [5, 6]. Hyperthermia, elevation of temperature inside tumor up to 40-42°C, is an effective modality for the treatment of cancer. Hyperthermia also acts as a radiation- and chemo- sensitizer. The heat inhibits reparation of free radical and increases the damage to the DNA of tumor cells. Furthermore, hyperthermia can increase perfusion and oxygenation of neoplastic hypoxic cells that may enhance tumor radiation response. The efficacy of radiotherapy concomitant with hyperthermia against head and neck cancer [7], melanoma [8], and breast cancer [9] were demonstrated. Moreover, hyperthermia enhances cytotoxicity of several anticancer agents, such as docetaxel, gemcitabine and 5-FU [10, 11]. The heat accelerates chemotherapeutics reactions, facilitates the uptake of drugs through cell membrane. Preclinical studies showed that the combination of 5-FU and hyperthermia could promote tumor cell apoptosis and increase the thermotolerance, consequently improves prognosis and reduces side effects of chemotherapy [12]. A few clinical trials of patients with head-and-neck squamous cell carcinoma with N_3_ cervical lymph node metastasis who were treated with chemoradiotherpy plus hyperthermia have been reported [7]. However, no trials using thermoradiation therapy plus systemic chemotherapy treated ESCC with supraclavicular lymph node metastasis have been reported. So the aim of present study was to evaluate the efficacy and toxicity of Intensity-modulated radiotherapy (IMRT) and hyperthermia in UMT-ESCC.

## RESULTS

From October 2006 and December 2013, a total of 50 UMT-ESCC patients with supraclavicular lymph node metastasis were enrolled in this study. The baseline clinicopathological characteristics are shown in Table 1. The study population had a median age of 58 years (range: 41-70 years). Most of the patients had 2 or more supraclavicular lymph node metastasis (27/50, 54.0%), and 46.0% (23/50) had solitary metastasis. 88.0% of the patients (44/50) had metastasis in unilateral side of supraclavicular, whereas 12.0% (6/50) had metastasis in bilateral side of supraclavicular. The median diameter of supraclavicular lymph node metastasis was 2.7 cm (range: 0.6-6.1 cm).

**Table 1:** Characteristics of 50 patients with UMT-ESCC

Characteristics	cases	%
Gender		
Male	44	88.0
Female	6	12.0
Age, years		
≤60	30	60.0
>60	20	40.0
Tumor location		
Upper thoracic	28	56.0
Middle thoracic	22	44.0
KPS		
>90	20	40.0
≤90	30	60.0
Tumor length		
≤5 cm	27	54.0
>5 cm	23	46.0
T stage		
T2	10	20.0
T3	21	42.0
T4	19	38.0
N stage		
N1	15	30.0
N2-3	35	70.0
supraclavicular lymph node metastasis		
Left	24	48.0
Right	20	40.0
Bilateral	6	12.0
Number of metastasis		
Solitary	23	46.0
Multiple	27	54.0
Maximum diameter of supraclavicular lymph node metastasis		
≤2.5 cm	23	46.0
>2.5 cm	27	54.0
Chemotherapy		
No	6	12.0
PF	12	24.0
DP	23	46.0
S1	9	18.0
Treatment response		
CR+PR	32	64.0
SD+PD	18	36.0

All patients received IMRT without any interruption. Of 50 patients enrolled, 36 patients received two cycles of chemotherapy. The other 9 patients only received one cycle of chemotherapy according to patients’ wish (5 patients), due to treatment-related toxicity (3 patients), due to reduced performance status (1 patient). Second cycle of chemotherapy delay occurred in 5 patients after the first cycle due to insufficient white blood cell counts or platelet counts. The median hyperthermia sessions were 8 (range: 2-12 sessions). Maximum temperature in the central surface of the supraclavicular lymph node was 43.3°C and minimum temperature was 42.3°C.

The efficacy of treatment according to chest and cervical CT and supraclavicular ultrasound was classified as CR in 12 cases (24.0%), PR in 20 cases (40.0%) and no change in 17 cases (34.0%). The disease control rate was 98.0% (49/50).

The treatment-related side effects are summarized in Table 2. No patients died as a result of treatment-related side effects and all the toxicities were manageable. The most common types of hematological toxicities were anemia (62.0%) and leukopenia (60.0%), but all the events were transient. The most common non-hematological toxicities were hyperthermia related pain (38.0%) and fatigue (40.0%). Most of these events were grade 1-2.

**Table 2:** Maximum toxicities observed during the treatment

Side effects	Grade 1	Grade 2	Grade 3	Grade 4
Hematological toxicities				
Anemia	18	13	0	0
Leukopenia	20	6	4	0
Thromocytopenia	8	5	2	0
Non-hematological toxicities				
Skin rash	9	2	1	0
Hyperthermia related pain	12	5	2	0
Fatigue	10	9	1	0
Nausea	15	5	0	0
Vomiting	6	3	0	0
Diarrhea	2	1	0	0

**Figure 1:**
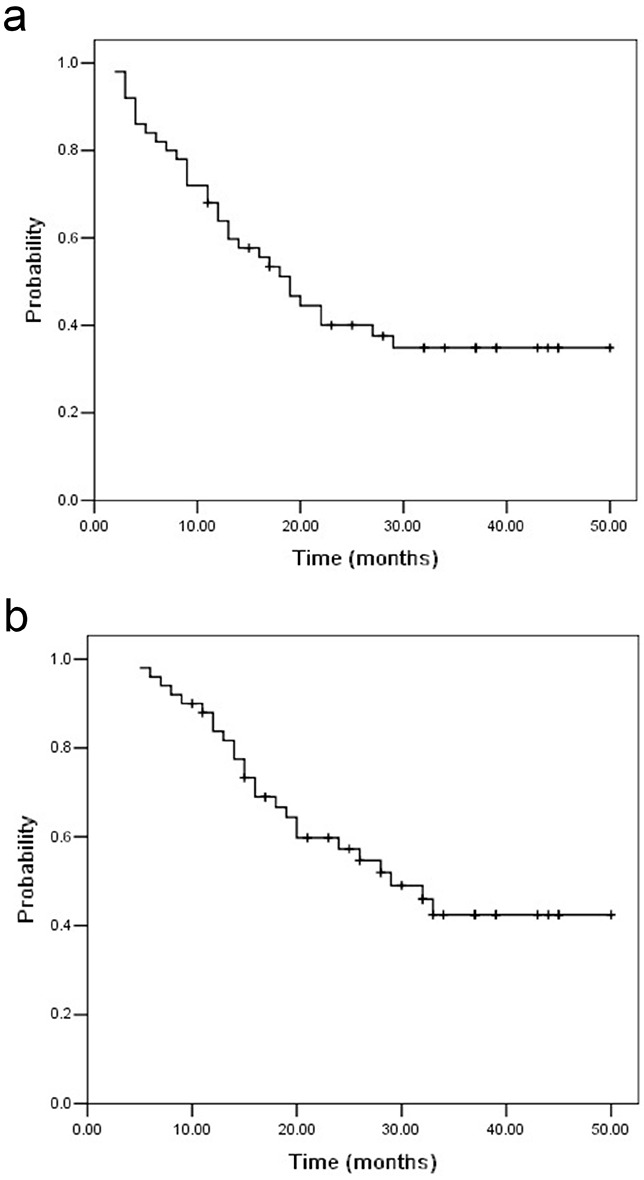
Progression-free survival a. and overall survival b. for UMT-ESCC patients with supraclavicular lymph node metastasis The median PFS and OS time were 19.0 months and 29.0 months.

**Figure 2:**
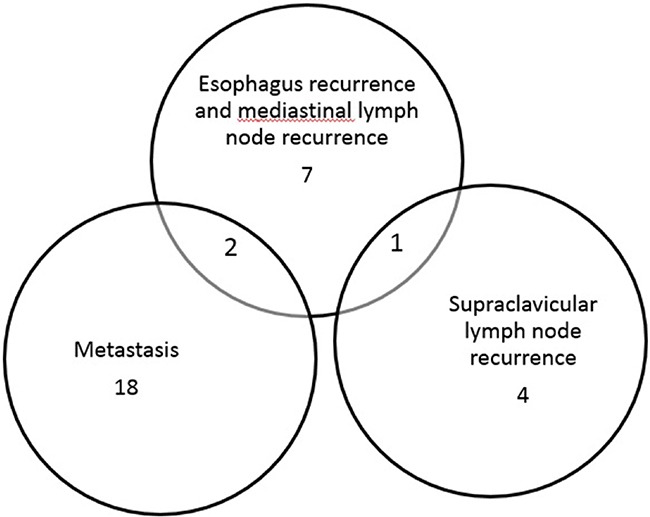
Venn diagram of the overlap among failure patterns after chemoradioherapy plus hyperthermia in UMT-ESCC patients with supraclavicular lymph node metastasis

The follow-up periods were 5.0-50.0 months (median22.0 months) in all patients. The median PFS and OS time were 19.0 months and 29.0 months. The 1-year, 2-year and 3-year PFS rate were 63.9%, 40.1% and 34.9% (Figure 1a), whereas the 1-year, 2-year and 3-year OS rate were 83.8%, 57.3% and 42.5% (Figure 1b). Thirty-one patients (62.0%) developed disease recurrence. Failure patterns for the entire population were demonstrated in Table 3 and Figure 2. Supraclavicular area control at 3-years was 83.8%, including 5 patients with supraclavicular lymph node recurrence. One patient developed a supraclavicular recurrence outside the hyperthermia field. Most of patients (36.0%) experienced distal organ metastasis.

**Table 3:** Failure patterns after chemoradioherapy plus hyperthermia in UMT-ESCC patients with supraclavicular lymph node metastasis

Failure pattern	n (%)	PFS time, months (Median, range)
Distant metastasis	18 (36.0)	10.0 (7.0-12.0)
Supraclavicular lymph node recurrence		
In field	4 (8.0)	16.0 (9.0-22.0)
Out field	1 (2.0)	4.0
Mediastinal lymph node metastasis	3 (6.0)	6.0 (5.0-9.5)
Esophagus recurrence	4 (8.0)	16.5 (7.0-17.0)
Local recurrence and distant metastasis	2 (4.0)	20.5

We performed univariate analysis for nine clinicopathological factors to find practically useful prognostic factors (Table 4). The clinicopathological factors included gender, age, KPS, T stage, tumor length, tumor location, chemotherapy, maximum diameter of supraclavicular lymph node metastasis and number of supraclavicular lymph node metastasis. Univariate analysis for PFS showed that KPS, tumor length, chemotherapy and N stage were four significant prognostic factors (KPS: HR=0.41, p=0.014; tumor length: HR=2.49, p=0.013; chemotherapy: HR=0.37, p=0.046; N stage: HR=9.54, p=0.002). Additionally, T stage was found to have a borderline statistically significant correlation with PFS (p=0.057). KPS, T stage, tumor length, number of supraclavicular lymph node metastasis and N stage were significant prognostic factors for OS (KPS: HR=0.45, p=0.049, T stage: HR=2.41, p=0.029, tumor length: HR=4.20, p=0.001; number of supraclavicular lymph node metastasis: HR=2.65, p=0.030; N stage: HR=6.95, p=0.009). Then we performed a multivariate analysis for these factors whose presence significantly affected prognosis (Table 4). The results showed that tumor length, chemotherapy and N stage were significantly related with PFS (tumor length: HR=2.63, p=0.022; chemotherapy: HR=0.15, p=0.002; N stage: HR=9.96, p=0.002). Cox regression also revealed that tumor length and N stage were only two independent predictors of OS (tumor length: HR=2.92, p=0.021; N stage: HR=8.07, p=0.019).

**Table 4:** Univariate analysis of PFS and OS among 50 patients with UMT-ESCC with supraclavicular lymph node metastasis

Variables	PFS				OS			
	Univariate analysis		Multivariate analysis		Univariate analysis		Multivariate analysis	
	HR	p	HR	p	HR	p	HR	p
Gender (Male Vs Femal)	0.80	0.682	-	-	0.87	0.826	-	-
Age (>60 Vs ≤60)	1.05	0.888	-	-	0.77	0.539	-	-
KPS (>90 Vs ≤90)	0.41	0.014	0.53	0.118	0.45	0.049	0.69	0.426
T stage (T_4_ Vs T_2-3_)	2.00	0.057	-	-	2.41	0.029	1.81	0.180
Tumor length (>5 cm Vs ≤5 cm)	2.49	0.013	2.30	0.043	4.20	0.001	3.65	0.008
Tumor location (Middle Vs upper)	0.65	0.249	-	-	0.72	0.435	-	-
Chemotherapy (Yes Vs no)	0.37	0.046	0.42	0.037	0.39	0.096	-	-
Maximum diameter of SLN metastasis (≥2.5 cm Vs <2.5 cm)	1.25	0.544	-	-	1.04	0.917	-	-
Number of SLN metastasis (Multiple Vs solitary)	1.70	0.152	-	-	2.65	0.030	1.03	0.964
N stage	9.54	0.002	9.96	0.002	6.95	0.009	8.07	0.019

## DISCUSSION

UMT-ESCC is one of the most common primary tumors in patients with supraclavicular lymph node metastasis. The rate of supraclavicular lymph node metastasis is up to 30-40% for all patients who underwent radical esophagectomy plus three-field lymph node dissection [13]. A combination treatment of hyperthermia and chemoradiotherapy improved overall survival in head and neck cancer patients with large cervical lymph node metastasis. In 1993, an Italian phase I-II study [14] investigated that patients with measurable neck metastases from previously untreated squamous cell head and neck cancer undergoing thermochemoradiotherapy. They reported a complete response rate of 72.2% (95% confidence interval 51-93.4%), 16.6% of PR and 11.1% of no change. A Japanese phase I study [15] conducted by Tohnai et al included 8 patients with oral cancer with N_3_ cervical lymph node metastasis. All the patients received neo-adjuvant thermochemoradiotherapy. Treatment consisted of hyperthermia (four-weekly sessions), daily concurrent radiation therapy (total 40 Gy) and cisplatin (5-10 mg/day, total dose: 100 mg/m^2^, 30 min before each session of radiotherapy). They reported that six patients experienced PR and 5-year overall survival rate is 70% in all patients. In a retrospective study conducted by Mitsudo et al [7], patients with oral cancer with N3 cervical lymph node metastasis experienced thermochemoradiation therapy using superselective intra-arterial infusion. Five-year survival and loco-regional control rates were 51% and 88%, respectively. Based on these findings, hyperthermia, and radiation in patients with cervical lymph node metastasis is promising and well tolerated with excellent local control and overall survival. In present study, supraclavicular hyperthermia combined with chemoradiotherapy resulted in median PFS of 19.0 months and median OS of 29.0 months, which was longer than that reported for chemoradiotherapy [16, 17]. The response rate was 64.0%. The 3-year PFS rate and OS rate were 34.9% and 42.5%, respectively. The supraclavicular lymph node recurrence probability was 8.0%, which is lower than that reported for chemoradiotherapy [17]. Patients with shorter primary tumor length had a better outcome than those with longer tumor length, which is consistent with the previous report [17]. Another finding in this study is that number of supraclavicular lymph node metastasis should be considered a prognostic factor in ESCC patients with supraclavicular lymph node metastasis. Patients with multiple supraclavicular lymph node metastasis had an elevated risk of death compared to patients with solitary supraclavicular lymph node metastasis. Thus we suppose that the patients with multiple supraclavicular lymph node metastasis may need supraclavicular hyperthermia because they are at higher risk for death.

It has been demonstrated that hyperthermia induced esophageal cancer cell apoptosis, due to the inhibition of survivin and the activation of caspase-3. In particular, the peak apoptosis can be reached at 43°C [18]. Moreover, constant incubating esophageal cancer cell at 43°C for 30 minutes can influence the cell cycle distribution and decrease the repair of sublethal damage [19]. Therefore, based on these research studies, this present study showed that the addition of hyperthermia to definitive chemoradiotherapy resulted in excellent locoregional control. The efficacy of regional hyperthermia has also been investigated in patients with esophageal cancer. In a phase II study [16], 28 patients treated with preoperative chemoradiotherapy combined with regional hyperthermia followed by esophageal resection. A pathologically CR rate was 19% and 3-year overall survival rate was 54.0%. In a retrospective study, preoperative chemoradiotherapy and hyperthermia can significantly improve patients’ overall survival versus chemoradiotherapy alone [17], with 5-year overall survival rate from 35.3% to 53.1%. However, there are a few studies on the combination of regional hyperthermia and definitive chemoradiotherapy for ESCC. In this present study, only supraclavicular metastatic lymph nodes were included in the heating area. Generally, hyperthermia inhibits the radiation-induced DNA damageand radioresistant cancer cells are thermosensitive. Metastatic lymph node in supraclavicular fossa tends to be broad and superficial, making it very suitable for heating with microwave device. On the other hand, esophageal normal tissue injuries, such as bleeding, congestion and perforation are recognized fatal complications of radiotherapy of ESCC with high radiation dose. The dosage of definitive radiotherapy for primary tumor is 60 Gy, a little bit higher than that of preoperative radiotherapy (45-50 Gy). Treatment modality in this area has been limited by the tolerance threshold of surrounding structures. In a previous study [16], 27 patients were treated with chemoradiotherapy plus hyperthermia. The dose of radiotherapy was only 41.4 Gy. During the treatment, one patients experienced massive esophageal bleeding and another patients experienced severe fever in several hours following the hyperthermia. The possible reason might be that hyperthermia combined with chemotherapy increased the risk of mucositis, which leading to a higher probability of esophageal bleeding and inflammation. From this perspective, we did not give hyperthermia to UMT-ESCC primary tumor. In the present study, all patients completed the chemoradiotherapy with supraclavicular hyperthermia. The most common type of toxicity was chemoradiotherapy related hematological toxicities, such as anemia and leukopenia. Regional hyperthermia -related pain was observed in 38.0% of patients and all pains were reversible. No fatal side effects were occurred during the chemoradiotherapy with supraclavicular hyperthermia.

Several studies have investigated that definitive chemoradiotherapy is an important and hopeful treatment option for patients with ESCC with supraclavicular lymph node metastasis (see Table 5). The 3-year PFS and OS in these studies were 6-20% and 12-33.5% respectively. The 3-year PFS and OS in present study were a bit higher than that in these studies. But all these studies (including present study) are phase I or II clinical trials. Thus, large randomized controlled clinical trials are needed to confirm it.

**Table 5:** Comparisons of definitive chemoradiotherapy in ESCC with supraclavicular or cervical lymph node metastasis

Authors	Year	Radiotherapy modality	Radiotherapy dose (Gy)	Chemotherapy regimens	Number of patients (n)	Toxicity	Survival	Pronostic factors
Liu et al. [20]	2011	3D-CRT	50-70	Cisplatin/5-fluorouracil	78	N.R	3 year OS rate: 15%.	Primary tumor location, tumor length, cervical nodal involvement.
Zhang et al. [21]	2014	3D-CRT	46-70	Cisplatin/5-fluorouracil or cisplatin/docetaxel	106	N.R	3 year PFS rate: 26.4% 3 year OS rate: 33.5%.	Gender, T stage, chemotherapy regimen and cervical lymph node involvement.
Zhang et al. [22]	2014	IMRT	Median: 60	Cisplatin/docetaxel	139	The most common grades 3 and 4 toxicities were leukopenia (48 patients; 34.5%) and gastrointestinal toxicity (15 patients; 10.8%). Laryngeal edema occurred in one patient.	3 year PFS rate: 20.1% 3 year OS rate: 27.9%.	Response to treatment, T stage, pathological grade, and cervical lymph nodal involvement.
Yamashita et al. [23]	2014	3D-CRT	50	Nedaplatin/5-fluorouracil	53	Acute grade 3-5 esophagitis and neutropenia was seen in 11% and 81%, respectively. Late grade 3-5 toxicity in esophagus was seen in 4%.	3 year PFS rate: 6% 3 year OS rate: 12%.	N.R

3D-CRT: Three-dimensional conformal radiotherapy; N.R: Not referred; OS: Overall survival; PFS: progression free survival; IMRT: intensity-modulated radiotherapy.

There are several limitations in this study. The major limitation is that not all the patients received the same anticancer treatment, including radiation exposure dosages, chemotherapy regimens, and chemotherapy cycles. Selection bias could not be avoided due to its retrospective study design. Owing to the small sample number, we must acknowledge the potential presence of type II error in our conclusion. Finally, tumor staging in this study was based on radiological examination.

In conclusion, despite the intrinsic limitation of a small analysis, our present study demonstrated that the combination of chemoradiotherapy with supraclavicular hyperthermia has low toxicity and well tolerated with excellent supraclavicular control in UMT-ESCC with supraclavicular lymph node metastasis. Randomized, well designed phase III clinical trial are needed to assess the efficacy of combined hyperthermia and chemoradiotherapy.

## MATERIALS AND METHODS

### Patient selection

The selective criteria included: newly confirmed to have UMT-ESCC and supraclavicular lymph node metastasis; no chemotherapy or radiotherapy previously; staged by endoscopic ultrasonography of the esophagus, computed tomography (CT) scan of the neck, chest and upper abdomen and ultrasonography of the supraclavicular lymph node region; supraclavicular lymph node metastasis confirmed by biopsy and histology; without distant organ metastasis; Karnofsky performance status (KPS) ≥70; adequate liver function (serum bilirubin ≤ 1.5 x ULN (Upper limits of normal), serum transaminases ≤ 2.5 x ULN); adequate renal function (serum creatinine ≤1.5 mg/dL, and blood urea nitrogen level ≤25 mg/dL); adequate bone marrow function (white blood cell count of at least 3500 cells/mm^3^, a platelet count of at least 100,000/mm^3^, and a hemoglobin level of at least 9.0 g/dL). Finally, a total of 50 eligible patients were enrolled in our retrospective study. Clinicopathologic information such as age, gender, tumor location, tumor stage, tumor length and supraclavicular lymph node status were obtained from patients’ charts.

### Radiotherapy delivery

All patients were immobilized in a supine position with the use of thermoplastic cast. Simulation CT images were acquired with 5 mm thickness throughout the entire neck and thorax. The gross tumor volume (GTV) was defined as esophageal cancer shown on the CT and mediastinal lymph nodes with the short axis of ≥10 mm and supraclavicular lymph nodes with the short axis of ≥10 mm. The clinical target volume (CTV) included the primary tumor with a 2.5 cm margin in the esophageal long axis superiorly and inferiorly, but 1.0 cm margin was added around GTV. The plan target volume (PTV) was defined as the CTV plus 0.5-1.0cm to account for the daily setup variation and respiratory movement. Radiation plans for intensity-modulated radiation therapy (IMRT) were generated using Pinnacle Version 8.0. IMRT was delivered using 6-MV X-rays (Radiotherapy machine: Varian Clinical 23EX) with multi-leaf collimator (MLC). A fractional daily dose of 1.8-2.0 Gy (5 days per week) was prescribed. The median delivered dose of IMRT was 60.0 Gy (ranged: 50.0-71.6Gy). The dose to the organs at risk was constrained as follows: The lung dose was limited to V_20_<30%, V_30_<20% and a mean dose less than 15 Gy. Spinal cord maximum dose was held to 45 Gy. The mean heart dose was ≤30 Gy.

### Chemotherapy delivery

Forty-four patients (88.0%) received concurrent chemoradiotherapy based on cisplatin regimens. The chemotherapy regimens consisted of three combinations: (1) 60 mg/m^2^ of paclitaxel on days 1 and day 22, and 25 mg/m^2^ of cisplatin on days 1-3 and days 22-24; (2) 25 mg/m^2^ of cisplatin on days 1-3 and days 22-24, and 24 h intravenous continuous infusion of 1000 mg/m^2^ of 5-fluorouracil (5-FU) on days 1 to 4 and days 22-26; (3) 25 mg/m^2^ of cisplatin on days 1-3 and days 22-24, and 60 mg/m^2^ of S1 twice a day on days 1-14 and days 22-35. Chemoradiotherapy was suspended if KPS reduced to < 60 or the white cell count decreased to less than 1 000/mL or hemoglobin decreased to less than 7.0 g/dL or the platelet count decreased to ≤ 50 000/ mm^3^.

### Hyperthermia delivery

Radiofrequency capacitive heating device, with microwave spiral strip applicators, named HRL-001 (Jilin Maida, China), maximum output at 800W, were used for external heating. Hyperthermia was delivered as soon as possible after the irradiation (usually within 30 min), or 2h after chemotherapy finished. The applicator was chosen depending on the surface of the superclavical to be treated and tumor extent. In the supine position, patients were positioned with a pillow under their heads and their arms resting at the side of their body. Ultra sound was used every two weeks to observe tumor extent, position and depth. Hyperthermia was confined to supraclavicular lymph node metastasis. The area of hyperthermia field depended on tumor extent. Normally, a gap of 2 cm was required between hyperthermia field and tumor. In case of bilateral supraclavicular nodal metastasis, two hyperthermia fields were used simultaneously. The objective of hyperthermia treatment was to achieve a minimal tumor temperature higher than 43.0°C at reference thermometer, while limiting maximum temperature of the adjacent skin at 43.5°C, and to maintain this condition for at least 45 min. The reference thermometer was defined as centrally located probe in relation to the tumor. Continuous temperature monitoring was performed by thermocouples in multi-sensor surface and interstitial probes. Patients were carefully instructed to report any discomfort during treatment. Heart rate, blood pressure, and peripheral oxygenation were automatically evaluated every 5 min.

### Treatment response and toxicity

During the chemoradiotherapy, patients received physical examination and upper gastrointestinal radiography once a week. Treatment response was evaluated on CT images, upper gastrointestinal radiography and upper GI endoscopy according to Response Evaluation Criteria in Solid Tumors (RECIST), version3.0. Furthermore, if there was suspicious of local recurrences, the patients also needed further upper GI radiography and endoscopy to confirm. The CR (complete response), PR (partial response), SD (stable disease) and PD (progressive disease) were assessed at an interval of 4-6 weeks after chemoradiotherapy to confirm the objective response. Treatment-related acute toxicity was scored by the Common Terminology Criteria for Adverse Events Version 3.0 once a week during treatment and then every 3 months at follow-up visits. Toxicity was recorded as the highest grade experienced during the whole treatment. All patients received standardized follow-up, 1 month after radiation and at 3-month intervals thereafter.

### Statistical analysis

PFS was defined as the duration from the date of first day of chemoradiotherapy to the disease progression. OS was calculated as the time from the date of first day of chemoradiotherapy to death or censoring. Survival curves were estimated by the univariate Kaplan-Meier method. The log-rank test was applied to check the significant differences in the curves among groups. The independent value of characteristics was evaluated in multivariate analysis using the Cox proportional hazard model. All statistical analyses were performed using SPSS 13.0 (Inc, Chicago, IL, USA). p values of < 0.05 were considered to indicate statistical significance.

## References

[1] Liu Q, Cai XW, Wu B, Zhu ZF, Chen HQ, Fu XL (2014). Patterns of failure after radical surgery among patients with thoracic esophageal squamous cell carcinoma: implications for the clinical target volume design of postoperative radiotherapy. PloS one.

[2] Li M, Liu Y, Xu L, Huang Y, Li W, Yu J, Kong L (2015). Computed tomography-based distribution of involved lymph nodes in patients with upper esophageal cancer. Current oncology.

[3] Huang W, Huang Y, Sun J, Liu X, Zhang J, Zhou T, Zhang B, Li B (2015). Atlas of the thoracic lymph nodal delineation and recommendations for lymph nodal CTV of esophageal squamous cell cancer in radiation therapy from China. Radiotherapy and oncology.

[4] Tachimori Y, Ozawa S, Numasaki H, Matsubara H, Shinoda M, Toh Y, Udagawa H (2014). and Registration Committee for Esophageal Cancer of the Japan Esophageal S. Supraclavicular node metastasis from thoracic esophageal carcinoma: A surgical series from a Japanese multi-institutional nationwide registry of esophageal cancer. The Journal of thoracic and cardiovascular surgery.

[5] Lu JY, Cheung ML, Huang BT, Wu LL, Xie WJ, Chen ZJ, Li DR, Xie LX (2015). Improving target coverage and organ-at-risk sparing in intensity-modulated radiotherapy for cervical oesophageal cancer using a simple optimisation method. PloS one.

[6] Gerber N, Ilson DH, Wu AJ, Janjigian YY, Kelsen DP, Zheng J, Zhang Z, Bains MS, Rizk N, Rusch VW, Goodman KA (2014). Outcomes of induction chemotherapy followed by chemoradiation using intensity-modulated radiation therapy for esophageal adenocarcinoma. Diseases of the esophagus.

[7] Mitsudo K, Koizumi T, Iida M, Iwai T, Oguri S, Yamamoto N, Itoh Y, Kioi M, Hirota M, Tohnai I (2012). Thermochemoradiation therapy using superselective intra-arterial infusion via superficial temporal and occipital arteries for oral cancer with N3 cervical lymph node metastases. International journal of radiation oncology, biology, physics.

[8] Overgaard J, Gonzalez Gonzalez D, Hulshof MC, Arcangeli G, Dahl O, Mella O, Bentzen SM (1996). Hyperthermia as an adjuvant to radiation therapy of recurrent or metastatic malignant melanoma. A multicentre randomized trial by the European Society for Hyperthermic Oncology. International journal of hyperthermia.

[9] Vernon CC, Hand JW, Field SB, Machin D, Whaley JB, van der Zee J, van Putten WL, van Rhoon GC, van Dijk JD, Gonzalez Gonzalez D, Liu FF, Goodman P, Sherar M (1996). Radiotherapy with or without hyperthermia in the treatment of superficial localized breast cancer: results from five randomized controlled trials. International Collaborative Hyperthermia Group. International journal of radiation oncology, biology, physics.

[10] Mohamed F, Marchettini P, Stuart OA, Urano M, Sugarbaker PH (2003). Thermal enhancement of new chemotherapeutic agents at moderate hyperthermia. Annals of surgical oncology.

[11] Maeta M, Sawata T, Kaibara N (1993). Effects of hyperthermia on the metabolism of 5-fluorouracil in vitro. International journal of hyperthermia.

[12] Liu T, Ye YW, Zhu AL, Yang Z, Fu Y, Wei CQ, Liu Q, Zhao CL, Wang GJ, Zhang XF (2015). Hyperthermia combined with 5-fluorouracil promoted apoptosis and enhanced thermotolerance in human gastric cancer cell line SGC-7901. OncoTargets and therapy.

[13] Chen J, Wu S, Zheng X, Pan J, Zhu K, Chen Y, Li J, Liao L, Lin Y, Liao Z (2014). Cervical lymph node metastasis classified as regional nodal staging in thoracic esophageal squamous cell carcinoma after radical esophagectomy and three-field lymph node dissection. BMC surgery.

[14] Amichetti M, Graiff C, Fellin G, Pani G, Bolner A, Maluta S, Valdagni R (1993). Cisplatin, hyperthermia, and radiation (trimodal therapy) in patients with locally advanced head and neck tumors: a phase I-II study. International journal of radiation oncology, biology, physics.

[15] Tohnai I, Hayashi Y, Mitsudo K, Shigetomi T, Ueda M, Ishigaki T (2002). Prognostic evaluation of preoperative thermochemoradiotherapy for N(3) cervical lymph node metastases of oral cancer. Oncology.

[16] Hulshof MC, Van Haaren PM, Van Lanschot JJ, Richel DJ, Fockens P, Oldenborg S, Geijsen ED, Van Berge Henegouwen MI, Crezee J (2009). Preoperative chemoradiation combined with regional hyperthermia for patients with resectable esophageal cancer. International journal of hyperthermia.

[17] Morita M, Kuwano H, Araki K, Egashira A, Kawaguchi H, Saeki H, Kitamura K, Ohno S, Sugimachi K (2001). Prognostic significance of lymphocyte infiltration following preoperative chemoradiotherapy and hyperthermia for esophageal cancer. International journal of radiation oncology, biology, physics.

[18] Qin S, Xu C, Li S, Wang X, Sun X, Wang P, Zhang B, Ren H (2015). Hyperthermia induces apoptosis by targeting Survivin in esophageal cancer. Oncol Rep.

[19] Cui YH, Liang HJ, Zhang QQ, Li SQ, Li XR, Huo XQ, Yang QH, Li WW, Gu JF, Hua QL, Lu P, Miao ZH (2012). Radiosensitivity enhancement by arsenic trioxide in conjunction with hyperthermia in the EC-1 esophageal carcinoma cell line. Asian Pac J Cancer Prev.

[20] Liu H, Lu L, Zhu Q, Hao Y, Mo Y, Liu M, Hu Y, Cui N, Rong T (2011). Cervical nodal metastases of unresectable thoracic esophageal squamous cell carcinoma: characteristics of long-term survivors after concurrent chemoradiotherapy. Radiother Oncol.

[21] Zhang P, Xi M, Zhao L, Li QQ, He L, Liu S, Shen J, Liu MZ (2014). Unilateral cervical nodal metastasis is an independent prognostic factor for esophageal squamous cell carcinoma patients undergoing chemoradiotherapy: a retrospective study. PLoS One.

[22] Zhang P, Xi M, Zhao L, Li QQ, He LR, Liu SL, Shen JX, Liu MZ (2014). Efficacy and prognostic analysis of chemoradiotherapy in patients with thoracic esophageal squamous carcinoma with cervical lymph nodal metastasis alone. Radiat Oncol.

[23] Yamashita M, Takenaka HY, Nakagawa K (2014). Semi-radical chemoradiotherapy for 53 esophageal squamous cell carcinomas with supraclavicular lymph node metastasis in a single institutional retrospective study. Hepatogastroenterology.

